# The Effect of *Bifidobacterium animalis* ssp. *lactis* HN019 on Cellular Immune Function in Healthy Elderly Subjects: Systematic Review and Meta-Analysis

**DOI:** 10.3390/nu9030191

**Published:** 2017-02-24

**Authors:** Larry E. Miller, Liisa Lehtoranta, Markus J. Lehtinen

**Affiliations:** 1 Miller Scientific Consulting, Inc., 1854 Hendersonville Road, #231, Asheville, NC 28803, USA; 2 DuPont Nutrition and Health, Sokeritehtaantie 20, Kantvik 02460, Finland; Liisa.Lehtoranta@dupont.com (L.L.); Markus.Lehtinen@dupont.com (M.J.L.)

**Keywords:** aging, *Bifidobacterium*, elderly, immunity, probiotic

## Abstract

Elderly people have increased susceptibility to infections and cancer that are associated with decline in cellular immune function. The objective of this work was to determine the efficacy of *Bifidobacterium* (*B.*) *animalis* ssp. *lactis* HN019 (HN019) supplementation on cellular immune activity in healthy elderly subjects. We conducted a systematic review of Medline and Embase for controlled trials that reported polymorphonuclear (PMN) cell phagocytic capacity or natural killer (NK) cell tumoricidal activity following *B. lactis* HN019 consumption in the elderly. A random effects meta-analysis was performed with standardized mean difference (SMD) and 95% confidence interval between probiotic and control groups for each outcome. A total of four clinical trials were included in this analysis. *B. lactis* HN019 supplementation was highly efficacious in increasing PMN phagocytic capacity with an SMD of 0.74 (95% confidence interval: 0.38 to 1.11, *p* < 0.001) and moderately efficacious in increasing NK cell tumoricidal activity with an SMD of 0.43 (95% confidence interval: 0.08 to 0.78, *p* = 0.02). The main limitations of this research were the small number of included studies, short-term follow-up, and assessment of a single probiotic strain. In conclusion, daily consumption of *B. lactis* HN019 enhances NK cell and PMN function in healthy elderly adults.

## 1. Introduction

Globally, elderly people represent the fastest growing population. Older individuals have typically weaker immune responses to vaccination and elevated risk for infections, certain autoimmune diseases, and cancer [1]. Many of these health risks are a consequence of declining immune function associated with the aging process, i.e., immunosenescence [2]. Traditional hallmarks of immunosenescence include adaptive immunity components such as lower number and/or proportions of peripheral blood naïve T cells (cluster of differentiation 8 (CD8+)), increased number of memory/effector cytotoxic T cells (CD8+) [3], as well as altered capacity of peripheral blood T cells to proliferate and secrete cytokines [1]. Furthermore, B-cell function and quantity appear to decline with age [4]. Age-related alterations have also been reported for innate immune system and specifically for function of neutrophils and natural killer (NK) cells. Neutrophils, which are important for early immune response for infections, show decreased chemotaxis, phagocytic activity, and declined superoxide generation in the elderly [5,6]. Polymorphonuclear (PMN) cells in the blood are mostly neutrophils (90%–95%), together with smaller fraction of eosinophils and basophils. The number of NK-cells increases with age, but their signaling efficiency, cytokine production, and up regulation of co-stimulatory molecules is suboptimal, leading to a net decrease in function [7,8,9]. It has been suggested that underlying defect of the declined innate cellular function in the elderly may be a result of decreased sensitivity of toll like receptor (TLR) signaling that is important in recognizing microbial structures [10]. These changes in immune function may compromise the early recognition and elimination of virus infected and malignant cells [2].

Gut microbiota plays an important role in immunosenescence and is influenced by physiological aging process, lifestyle, and diet [11,12,13,14]. It has been shown that aging gut microbiota has specific features compared to microbiota of younger adults—like lower levels of bifidobacteria and higher levels of *Bacteroidetes* spp. [12,14]. The above changes in microbiota composition may be indicative of dysbiosis and poorer health. For instance, lower bifidobacteria levels have been found to associate with increased risk of *Clostridium difficile* associated diarrhea [15], hospitalization [16], antibiotic treatment [17], and frailty [18]. Thus, targeted dietary interventions that restore composition of the microbiota could reduce the risk of age related morbidities and improve the quality of the life of the elderly.

It has been suggested that probiotic bacteria could have potential for improving immune system function in the elderly [19]. Probiotics are known to interact with TLRs and other microbial pattern recognition receptors on immune system cells in intestinal mucosa and thus directly influence their functions [20]. Furthermore, specific probiotic strains may induce beneficial changes in the gut microbiota that have an impact on immune status. For instance, *B. longum* strains induced changes in the bifidobacteria population, which correlated with tumor necrosis factor (TNF)-α and interleukin (IL)-10 levels in plasma [21], and *Lactobacillus (L.) casei* and *L. plantarum* strains improved influenza virus vaccination responses in the elderly [22,23]. Although there are some clinical trials investigating probiotics and their effectiveness in improving immune responses in the elderly [19], systematic information on strain-specific effects of probiotics on the immunity in this population is lacking. One of the probiotics applied in clinical studies in this area is *Bifidobacterium (B.) animalis* ssp. *lactis* HN019. The objective of this systematic review and meta-analysis of controlled studies was to evaluate the effect of *B. lactis* HN019 versus non-probiotic control on cellular innate immune activity of healthy elderly subjects.

## 2. Materials and Methods

### 2.1. Literature Search

The study was performed according to the Preferred Reporting Items for Systematic Reviews and Meta-Analyses (PRISMA) [24]. Medline and Embase databases were searched for randomized or non-randomized controlled studies published in English-language journals that reported PMN cell phagocytosis activity or NK cell tumoricidal activity following *B. lactis* HN019 consumption in reportedly healthy, elderly (≥60 years) adults. No date restrictions were applied to the searches. The details of the Medline search strategy are listed in Table 1. The syntax for Embase was similar, but adapted as necessary. Additionally, manual searches were conducted using the Directory of Open Access Journals, Google Scholar, and the reference lists of included papers and relevant meta-analyses. The final search was conducted in June 2016.

### 2.2. Study Selection

One reviewer selected studies for inclusion in the review. Articles were then independently assessed by a second reviewer, who confirmed eligibility. Titles and abstracts were initially screened to exclude non-English manuscripts, review articles, commentaries, letters, case reports, animal studies, and obvious irrelevant studies. Full-texts of the remaining articles were retrieved and reviewed.

### 2.3. Data Extraction

Data were independently extracted from eligible articles by two reviewers. Data extraction discrepancies between the reviewers were resolved by consensus. The types of data recorded in the standardized data extraction forms included general manuscript information, subject characteristics, study characteristics, PMN phagocytic capacity, and NK cell tumoricidal activity. Potential sources of bias were assessed by evaluating randomization, blinding, type of control, and main outcome definitions among studies.

### 2.4. Data Synthesis

A random effects meta-analysis model was developed based on the a priori assumption that treatment effects would be heterogeneous among studies. For each outcome, a pooled standardized mean difference (SMD) and 95% confidence interval were calculated. For reference, SMD values of 0.2, 0.5, 0.8, and 1.0 are defined as small, medium, large, and very large effect sizes, respectively [25]. Forest plots were used to illustrate individual study findings and pooled meta-analysis results. Heterogeneity of effects across studies was planned to be estimated using the *I*^2^ statistic if at least 10 studies were included in the meta-analysis. *p*-values were two-sided with a significance level <0.05. Statistical analyses were performed using Comprehensive Meta-analysis version 2.2 (Biostat, Englewood, NJ, USA).

## 3. Results

**Figure 1:**
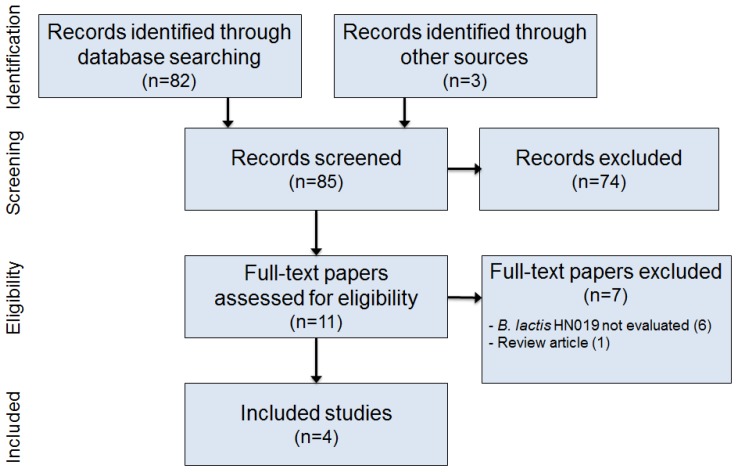
Preferred Reporting Items for Systematic Reviews and Meta-Analyses (PRISMA) study flow diagram.

The initial database search retrieved 82 titles and abstracts; hand searching relevant bibliographies identified three additional records. After screening records for inclusion criteria, 74 records were deemed irrelevant and 11 full text articles were reviewed for eligibility. Ultimately, four studies [26,27,28,29] were included in the final analysis. A flow diagram of study identification and selection is shown in Figure 1.

Regarding the evaluation of *B. lactis* HN019 relative to control, one study [28] was a randomized trial with a parallel control group and three studies [26,27,29] used a 3-week run-in period as the control. Subjects in each study were healthy elderly adults with a median age between 60 and 70 years. The interventions evaluated were daily consumption of low-fat milk, with or without *B. lactis* HN019. The daily dosages of *B. lactis* HN019 ranged from 5 × 10^9^ to 3 × 10^11^ colony forming units (cfu) and the treatment durations ranged from 3 to 6 weeks (Table 2). An assessment of potential sources of bias are listed in Table 3. One study [28] utilized random allocation and two studies [26,28] blinded subjects to the allocated intervention. The main outcome definitions were consistent across studies and, therefore, pooling data among studies was appropriate.

**Figure 2:**
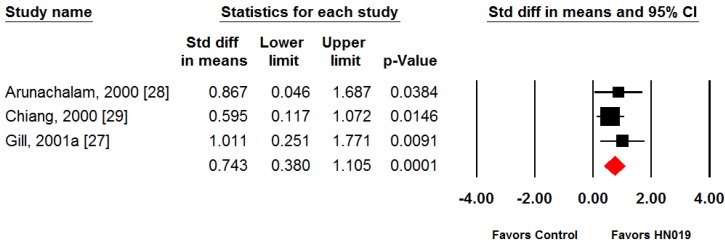
Forest plot of polymorphonuclear phagocytic capacity with consumption of *B. lactis* HN019 vs. control. Random effects meta-analysis using the standardized mean difference (SMD) statistic. The SMD of *B. lactis* HN019 relative to control is plotted for each study. A pooled estimate of SMD (diamond) and 95% confidence interval (diamond width) summarizes the effect size. Effects to the left of 0 indicate greater polymorphonuclear (PMN) phagocytic capacity with control; effects to the right of 0 indicate greater capacity with *B. lactis* HN019. When the horizontal bars of an individual study, or the pooled diamond width, cross 0, the effect is not significantly different. The pooled SMD was 0.74 (95% CI: 0.38 to 1.11, *p* < 0.001), representing a large treatment effect in favor of *B. lactis* HN019.

Phagocytic capacity outcomes were consistent among individual studies (SMD range: 0.60 to 1.01). Furthermore, each study reported statistically significant improvements in PMN phagocytic capacity with *B. lactis* HN019 relative to control. The pooled SMD for PMN phagocytic capacity was 0.74 (95% CI: 0.38 to 1.11, *p* < 0.001), representing a large treatment effect in favor of *B. lactis* HN019 (Figure 2). Heterogeneity in phagocytic capacity was not formally assessed given the small number of studies.

**Figure 3:**
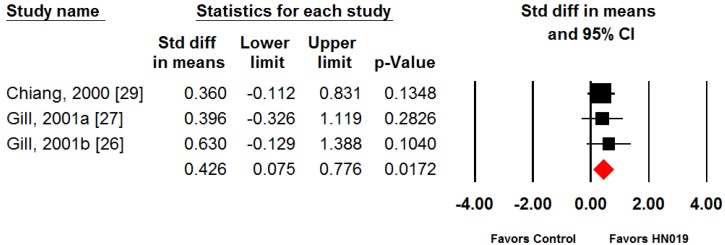
Forest plot of natural killer (NK) cell tumoricidal activity with consumption of *B. lactis* HN019 vs. control. Random effects meta-analysis using the standardized mean difference (SMD) statistic. The SMD of *B. lactis* HN019 relative to control is plotted for each study. A pooled estimate of SMD (diamond) and 95% confidence interval (diamond width) summarizes the effect size. Effects to the left of 0 indicate greater NK cell tumoricidal activity with control; effects to the right of 0 indicate greater activity with *B. lactis* HN019. When the horizontal bars of an individual study, or the pooled diamond width, cross 0, the effect is not significantly different. The pooled SMD was 0.43 (95% CI: 0.08 to 0.78, *p* = 0.02), representing a moderate treatment effect in favor of *B. lactis* HN019.

NK cell tumoricidal activity outcomes were consistent among individual studies (SMD range: 0.36 to 0.63). Although no individual study reported statistically significant differences between the groups, the pooled SMD for NK cell tumoricidal activity was 0.43 (95% CI: 0.08 to 0.78, *p* = 0.02), representing a statistically significant moderate treatment effect in favor of *B. lactis* HN019 (Figure 3). Heterogeneity in NK cell tumoricidal activity was not formally assessed given the small number of studies.

## 4. Discussion

With modern life-style the human life expectancy is increasing and the health in old age is of increasing concern to individuals and society. It has been suggested that probiotic supplementation could offer means to reverse some age-related changes in intestinal microflora composition and to help maintain the aging immune system that are associated with age-related morbidities [30]. Despite the potential benefits of the probiotics for the elderly, the efficacy of only few probiotic strains on immune function in this population has been tested in clinical studies. This systematic review and meta-analysis reports the efficacy of *B. lactis* HN019 on PMN phagocytic capacity and NK cell tumoricidal activity in healthy elderly subjects. The final analysis included four controlled clinical trials with variable designs, doses, and treatment durations. The outcomes in these studies included either PMN phagocytic capacity or NK cell tumor killing activity or both. The pooled data showed that short-term, 3 to 6 weeks, consumption of *B. lactis* HN019 resulted in significantly enhanced PMN phagocytic capacity and NK cell tumoricidal activity in the healthy elderly population.

Three independent clinical studies assessing the effect of *B. lactis* HN019 on PMN phagocytic capacity were included in this study [27,28,29]. Although the number of subjects in the trials was modest (Table 2), all consistently showed statistically significant effect (*p* < 0.05) on phagocytic capacity. The meta-analysis of the trials showed that HN019 had a large treatment effect (*p* < 0.001) on phagocytic capacity of PMN cells (Figure 2), confirming the original study findings. Whether the improvement in PMN function by HN019 leads to better resistance to infections was not studied in the original publications. However, it is recognized that phagocytosis is a first-line defense function of PMN cells against infectious diseases that are found more prevalent in the elderly population [31,32].

Previously published studies on the effect of probiotics on phagocytosis have reported that daily consumption of *B. lactis* Bi-07 improved the phagocytic activity of monocytes and granulocytes in healthy elderly adults [33], but in contrast, supplementation with *L. johnsonii* La1 decreased phagocytic activity of neutrophils in the elderly [34]. As intestinal bifidobacteria count decreases with age [12,14], it is noteworthy that two different bifidobacteria strains Bi-07 and HN019 were efficacious whereas the lactobacillus strain was not, however, the data is very limited to make any conclusions on the differences in the efficacy on the genera level. In fact, a recent consensus paper concluded that the effects of probiotics on immune function are strain-specific [35].

Whereas PMN phagocytosis and NK-cell cytotoxicity are key immune functions against infections, NK cells function also in the elimination of cancerous cells. This study included three clinical trials that investigated the effect of *B. lactis* HN019 on NK-cell tumoricidal activity, but none showed statistically significant results in the original publications [26,27,29]. However, the group sizes were relatively small and pooling the data in this meta-analysis resulted in a statistically significant (*p* = 0.017) treatment effect for improving NK-cell activity in the elderly (Figure 3). Although the result shows improvement in NK-cell activity, it remains to be studied if the improved function by *B. lactis* HN019 results in health benefit for the elderly. Nevertheless, research in the elderly shows that low NK cell activity is associated with the development of infectious diseases [7,36]. Also, the incidence of cancer and mortality rate has been found higher in populations with a low NK cell activity compared with those with higher NK cell activities [36,37,38].

Effect of other probiotics than HN019 studies have also been investigated on NK cell activity. A combination of *L. rhamnosus* HN001 and *L. acidophilus* NCFM resulted in a significantly increased cytotoxicity of the NK cells [39]*.* In contrast, *B. longum* BB536 intake for 12 weeks had no effect on NK cell activity in elderly patients fed by enteral tube feeding [40] and *L. gasseri* TMC0356 supplementation for 4 weeks had no significant impact on NK cell counts or NK cell activity [41]. Altogether three studies have assessed the effect of *L. casei* Shirota on NK cell function: (i) Takeda and colleagues showed that *L. casei* Shirota may elevate NK cell activity after 4 weeks when compared to placebo [42]; (ii) the consumption of *L. casei* Shirota was associated with a significant increase in NK cell activity relative to baseline, however the difference was not significant when compared with placebo [43]; and (iii) a study showed that NK-cell activity was not significantly augmented by a 3-week intake of *L. casei* Shirota [44]. In summary, the results on probiotic efficacy on NK cell activity in the elderly vary and may be strain and study dependent.

Although the impact of *B. lactis* HN019 on specific cellular immune parameters seems relatively clear there were still limitations inherent in the findings of this meta-analysis, including a potential for bias due to relatively small study populations. In addition, the study outcomes may have been affected by other factors such as the length of intervention, the formula, and the supplementation dose. In the studies included in this meta-analysis, the *B. lactis* HN019 supplementation was given to healthy elderly adulty for 3–6 weeks suggesting that relatively short term consumption of *B. lactis* HN019 has an impact on immune cell function in blood; however, it remains unknown if the stimulatory effect would have been maintained in longer term consumption [45]. The daily *B. lactis* HN019 doses were between 5 × 10^10^ and 3 × 10^11^ cfu/day in PMN studies and 5 × 10^9^–5 × 10^10^ cfu/day in NK cell studies, suggesting that HN019 is efficacious with a range of doses typically used in dietary supplements. In all the *B. lactis* HN019 clinical trials low-fat milk was used as a delivery vehicle, and as such, no firm conclusions on the role of the vehicle on these immune outcomes can be drawn. A further consideration relates to type of control. The study by Arunachalam et al. [28] used a parallel group assigned to placebo, whereas the other three studies [26,27,29] utilized a run-in period control. Due to a small number of included studies, subgroup analysis and meta-regression to investigate sources of heterogeneity in outcomes (e.g., type of control, study duration, patient age) was not possible. Finally, since this report was focused on a single probiotic strain, the results cannot be generalized to probiotics as a whole.

## 5. Conclusions

The findings from this meta-analysis suggest that daily short-term consumption of probiotic *B. lactis* HN019 enhances PMN phagocytic capacity and NK cell tumoricidal activity in healthy elderly adults. As the health of the elderly was not assessed in the original studies, the correlation between *B. lactis* HN019 mediated PMN and NK cell activity improvement and resistance to infection and diseases remains to be confirmed in future trials.

## Figures and Tables

**Table 1:** Medline search strategy.

Intervention Search Terms
1. Probiotic
2. Synbiotic
3. Bifidobacteri *
4. Lactis
5. B. lactis
6. HN019
7. Yogurt (yoghurt)
8. Fermented milk
**Outcomes Search Terms**
9. Phagocyt *
10. Polymorphonuclear
11. PMN
12. Natural killer cell
13. NK cell
14. Tumoricidal
15. Immun *
**Combination Terms**
16. or/1–8
17. or/9–15
18. and/16–17

An asterisk represents a wildcard symbol used in a search query to represent end truncation. NK: natural killer; PMN: polymorphonuclear.

**Table 2:** Study characteristics.

Study	No. Subjects (HN019:Control)	Female (%)	Age (Median, Range)	Delivery Vehicle	HN019 Daily Dose (cfu)	Intervention Duration ^c^
Arunachalam, 2000 [28]	13:12	72	69 (60–83)	Low-fat milk	3 × 10^11^	6 weeks
Chiang, 2000 [29]	27 ^a^	70	60 (41–81)	Low-fat milk	5 × 10^10^	3 weeks
Gill, 2001a [27]	15 ^a,b^	60	69 (63–84)	Low-fat milk	5 × 10^10 b^	3 weeks
Gill, 2001b [26]	14 ^a^	57	70 (60–84)	Low-fat milk	5 × 10^9^	3 weeks

cfu: colony forming units. ^a^ All subjects completed run-in control and *B. lactis* HN019 intervention; ^b^ Study randomized subjects to high dose (5 × 10^10^ cfu) or low dose (5 × 10^9^ cfu) *B. lactis* HN019; data from high-dose group only were used for analyses; ^c^ Represents duration of each intervention, not duration of entire study.

**Table 3:** Bias assessment.

Study	Randomization	Blinding	Control	Definitions	
				Phagocytic Capacity	NK Cell Tumoricidal Activity
Arunachalam, 2000 [28]	Yes	Yes	Parallel group	Relative increase in % PMN cells showing phagocytic activity	--
Chiang, 2000 [29]	No	No	3-week run-in period	% PMN cells showing phagocytic activity	% NK cell tumor killing activity
Gill, 2001a [27]	No	No	3-week run-in period	% PMN cells showing phagocytic activity	% NK cell tumor killing activity
Gill, 2001b [26]	No	Yes	3-week run-in period	--	% NK cell tumor killing activity

Dashed line indicates data not reported; NK: natural killer; PMN: polymononuclear.

## Supplementary Materials

The supplementary materials are available online at http://www.mdpi.com/2072-6643/9/3/191/s1.
